# Addressing Ethical Issues in Biomedical and Dental Publishing

**DOI:** 10.1055/s-0046-1820079

**Published:** 2026-04-24

**Authors:** Luca Fiorillo

**Affiliations:** 1 Department of Medicine and Surgery, University of Enna “Kore,” Enna, Italy; 2 Department of Dental Research Cell, Dr. D. Y. Patil Dental College and Hospital, Dr. D. Y. Patil Vidyapeeth (Deemed to be University), Pimpri, Pune, India

## Introduction

Scientific integrity is the cornerstone of academic research, yet recent trends have highlighted significant misconduct issues within dental publications. The integrity of scientific research is fundamental to the advancement of knowledge and the betterment of society. However, the academic community has been increasingly confronted with instances of research misconduct, which undermine public trust and can have detrimental consequences, particularly in the medical and dental fields. 1 A meta-analysis of biomedical research, including medicine and dentistry, found an alarming rate of misconduct in recent non-self-reported studies, underscoring the pervasiveness of this issue. High-profile cases have brought attention to the severity of scientific fraud. For instance, the retraction of a study promoting hydroxychloroquine as a coronavirus disease 2019 treatment underscored significant ethical breaches in research methodology and the importance of rigorous peer review. 2 Similarly, the case of Dr. Eliezer Masliah, a prominent neuroscientist found to have engaged in research misconduct, illustrates that such issues can permeate even esteemed institutions. The repercussions of scientific misconduct are profound, leading to misinformation, wasted resources, and potential harm to patients. Addressing these challenges requires a multifaceted approach, including developing robust ethical guidelines, implementing stringent oversight mechanisms, and promoting a culture that values integrity over output. 3 By confronting these issues head-on, the academic community can work toward restoring trust and ensuring that scientific research fulfills its role in advancing human health and knowledge. 4 These malpractices include citation doping, non-peer-reviewed journals, predatory publishing, and the emerging misuse of artificial intelligence (AI) in research processes. 5 6 Therefore, the objective of this Editorial is threefold: (1) to critically analyze the principal ethical distortions currently affecting biomedical and dental publishing; (2) to contextualize their specific implications within evidence-based dentistry; and (3) to propose strategic, forward-looking recommendations aimed at strengthening transparency, accountability, and responsible AI integration in dental research ecosystems.

## Conceptual Framework and Sources

The article draws on peer-reviewed literature on research misconduct, citation manipulation, predatory publishing, authorship irregularities, bibliometric distortions, and the misuse of AI in the biomedical and dental sciences. Key case studies, international guidelines (e.g., research integrity frameworks), and documented retraction events were examined to identify recurring patterns and systemic vulnerabilities. The objective was not quantitative synthesis but conceptual integration, with particular emphasis on implications for dental research and clinical translation.

## Results

### Citation Doping and Self-Citations

- Self-citations and citation requests for nonrelevant works undermine the credibility of research outputs.
- Establish guidelines to limit self-citations and discourage requesting citations of irrelevant works. 7 8

### Predatory Journals

- Predatory journals exploit researchers by charging publication fees without providing legitimate peer review or editorial services.
- Educate researchers on identifying predatory journals using resources like Beall's List and promote the use of reputable open-access journals. 9

### AI Misuse in Research and Publication

- The misuse of AI in data generation, analysis, and manuscript writing can lead to inaccurate or fraudulent research outputs. Instances include AI-generated data fabrication and AI tools to create misleadingly polished manuscripts.
- Develop ethical guidelines for the appropriate use of AI in research, emphasizing transparency and accountability. Encourage the use of AI tools to enhance research quality while ensuring human oversight and validation. 7

### Ethical Guidelines

- The German Rectors' Conference and other academic bodies have set balanced guidelines emphasizing good research practices. 10
- Develop similar guidelines tailored to the dental research community, focusing on rewarding ethical research and penalizing fraudulent activities. 11

## Discussion

Addressing scientific misconduct and AI misuse is complex and requires a comprehensive strategy. Emphasis should be placed on creating an ethical research culture rather than merely penalizing misconduct. This includes fostering an environment where quality research is recognized and predatory practices are discouraged. 12 13 Patterns of strategic co-authorship and fabricated or irrelevant citations undermine the validity of bibliometric indicators and the credibility of the scholarly record. Gift/guest and ghost authorship dilute accountability and misrepresent contributorship, while paper mills and predatory outlets amplify “fake citations” and propagate nonreproducible claims. Coercive citation practices—by editors/reviewers or within tight co-author networks—can artificially inflate metrics without advancing knowledge, and have been empirically documented across disciplines. Conceptual taxonomies of self-citation (direct, co-author, collaborative) clarify how citation rings and reciprocal agreements distort impact measures. Practical safeguards include strict adherence to contributorship criteria (with transparent statements), systematic checks of reference relevance during peer review, screening for anomalous citation clusters, and routine audits of retraction-linked citations—measures that reduce gaming while preserving legitimate scholarly influence. Additionally, integrating ethical use of AI into research guidelines is crucial to prevent misuse and ensure the validity of research outcomes. As a famous song reports, we are probably moving from virtual reality to virtual insanity.

“Amicus Plato, sed magis amica veritas.” *(I am a friend of Plato, but a greater friend of truth.)* — *Aristotle*

The issues outlined in this Editorial highlight the multifaceted challenges that threaten the integrity of scientific research, especially in dentistry. These challenges, from predatory publishing and paper mills to ghost authorship, bibliometric manipulation, and AI misuse, pose a significant risk to the credibility, reliability, and ethical foundations of academic work. 14 This discussion aims to synthesize these issues and propose actionable strategies for the academic community. The proliferation of predatory journals exemplifies a key vulnerability in the publishing ecosystem. These journals exploit researchers' pressures, such as the need to publish for career advancement, by offering rapid publication without rigorous peer review. This undermines evidence-based practices, particularly in dentistry, where unverified research directly impacts clinical decisions and patient outcomes. 15 Evidence has shown that these journals often fail to meet minimal methodological and ethical standards, as initially warned by pioneers of evidence-based medicine like David Sackett. 15 The need for heightened scrutiny in journal selection, embodied by frameworks like “Think, Check, Submit,” becomes critical to combating this trend. 16 Paper mills further erode academic integrity by introducing fabricated data and fictitious authorship into the literature. The scale of this issue is underscored by Wiley's 2024 retraction of over 11,000 papers linked to such practices, a move that highlights the widespread infiltration of fraudulent work into even reputable journals. The economic ramifications and the reputational damage to the publishing industry call for stricter editorial policies and technological tools to identify and eliminate fraudulent submissions. 17 Recent publisher investigations have documented thousands of retractions linked to paper mill activities, highlighting the industrial scale of fabricated or manipulated submissions. Similarly, meta-research studies have reported measurable rates of coercive citation requests and inflated self-citation patterns across disciplines, confirming that bibliometric manipulation is not anecdotal but structurally embedded in certain academic environments. 18 19 20

Ghost authorship introduces another layer of ethical concern by misrepresenting research contributions. The prevalence of honorary and ghost authorship in high-impact journals compromises transparency and accountability. This practice distorts the scholarly record and diminishes the value of genuine contributions, highlighting the necessity for clear authorship criteria and strict enforcement by journals and institutions. 21 The misuse of bibliometric parameters, particularly self-citation manipulation, exemplifies the dangers of over-reliance on quantitative metrics in academia. The introduction of the Fi-Index offers a novel solution, providing a quantitative measure to evaluate the impact of self-citation on an author's h-index. Tools like the Fi-Index could mitigate the misuse of bibliometrics and encourage more ethical research practices by providing a transparent and data-driven approach to assessing citation practices. 22 The emergence of AI in academic publishing introduces both opportunities and challenges.

While AI tools can enhance manuscript drafting and peer review efficiency, their misuse risks undermining ethical standards. The potential for plagiarism, bias reinforcement, and reduced analytical rigor necessitates developing ethical guidelines to ensure transparency and accountability. Researchers and editors must disclose AI usage and maintain human oversight in critical evaluations to prevent ethical breaches. These unethical dental and medical research practices can have severe consequences, mainly through the “multiplication of patients” phenomenon. 23 24 The fabrication or duplication of patient data to achieve desired sample sizes jeopardizes the validity of clinical recommendations, potentially harming patients. This underscores the urgent need for robust ethical oversight and stringent methodological standards in clinical research. The broader implications of these challenges are profound. The rapid proliferation of predatory practices and ethical breaches threatens to erode the foundational principles of evidence-based science. 15 This issue is particularly pronounced in dentistry, where compromised research can obscure valuable findings and mislead clinical practices. A study by Valente illustrates the scale of this problem, with only a fraction of journals meeting credible indexing standards. 25 To address these challenges, a multi-pronged approach is essential. Education and awareness campaigns must equip researchers with the tools to identify and avoid predatory practices. Policy reforms should mandate transparency in AI usage, discourage bibliometric manipulation, and enforce stringent authorship criteria. 26 Technological solutions, such as AI-driven systems to detect misconduct, could complement these efforts by flagging citation patterns and submission irregularities. 27 28

By fostering collaboration, transparency, and ethical accountability, the academic community can safeguard the credibility of its work. Upholding the highest standards of excellence is imperative to ensuring that science remains a reliable foundation for clinical practice and societal progress.

## Conclusion

The ethical challenges facing biomedical and dental publishing extend beyond isolated cases of misconduct; they reflect systemic pressures linked to performance metrics, academic competition, and rapid technological transformation. Citation manipulation, predatory publishing, authorship distortion, and AI misuse collectively threaten the epistemic reliability of dental research and, by extension, patient safety.

A forward-looking strategy must therefore integrate educational initiatives, stricter editorial screening mechanisms, transparent AI disclosure policies, bibliometric auditing tools, and internationally harmonized ethical standards tailored to dental sciences. Continuous monitoring, cross-institutional collaboration, and adaptive regulatory frameworks will be essential to preserving the credibility of scholarly communication in the digital era. Research integrity must remain a shared responsibility among authors, reviewers, editors, institutions, and publishers.
